# The impact of temperature and insect-specific viruses on the transmission of alphaviruses by *Aedes japonicus japonicus*

**DOI:** 10.1128/spectrum.02668-24

**Published:** 2025-04-30

**Authors:** Stephanie Jansen, Dániel Cadar, Jana Christina Hey, Michelle Helms, Unchana Lange, Balázs Horváth, Hanna Jöst, Wolf-Peter Pfitzner, Jonas Schmidt-Chanasit, Renke Lühken, Anna Heitmann

**Affiliations:** 1 Bernhard Nocht Institute for Tropical Medicine 14888, Hamburg, Hamburg, Germany; 2 Faculty of Mathematics, Informatics and Natural Sciences, University of Hamburg 98889 , Hamburg, Hamburg, Germany; 3 Kommunale Aktionsgemeinschaft zur Bekämpfung der Schnakenplage e. V. (KABS), Speyer, Germany; National Microbiology Laboratory, Winnipeg, Manitoba, Canada

**Keywords:** *Aedes japonicus japonicus*, chikungunya virus, Sindbis virus, western enquine encephalitis virus, insect-specific virus, vector competence

## Abstract

Arthropod-borne virus (arbovirus) infections are increasing globally, and invasive mosquito species are spreading. Since the end of the last century, *Aedes japonicus* has continued to spread in Europe as well as in North America. *Aedes japonicus* is known to be able to transmit several viruses, but extensive information about the vector competence of *Ae. japonicus* for alphaviruses is missing. Therefore, we infected field-caught *Ae. japonicus* mosquitoes from Germany with different alphaviruses that occur in areas with either tropical or moderate temperatures and are clustered as arthritogenic or encephalitic alphaviruses. Additionally, we studied the influence of temperature and natural infections with insect-specific viruses (ISVs) on the vector competence of *Ae. japonicus*. Transmission of chikungunya virus was exclusively observed at the high-temperature profile of 27° ± 5°C, with a low transmission rate of 2.9%. Transmission of Sindbis virus and western equine encephalitis virus was observed at all investigated temperature profiles with higher transmission rates of 32%–57%. We identified seven different ISVs in the investigated *Ae. japonicus* mosquitoes, showing that coinfections with ISVs are very common. The interplay between arbovirus infections and concurrent multiple ISV infections is highly complex, and additional research is required to fully elucidate the detailed mechanisms underlying the outcomes of this study.

**IMPORTANCE:**

The spread of invasive mosquito species like *Aedes japonicus* poses a significant public health risk, particularly in the context of rising global temperatures and the growing prevalence of arbovirus infections. This study provides critical insights into the ability of *Aedes japonicus* to transmit alphaviruses such as chikungunya, Sindbis, and western equine encephalitis under different temperature conditions. The identification of multiple insect-specific viruses co-infecting the mosquitoes highlights the complexity of arbovirus transmission and underscores the need for further research. Understanding the interplay between environmental factors like temperature and viral coinfections is essential for predicting and mitigating future outbreaks. This work advances our knowledge of vector competence, which is helpful for developing strategies for risk assessment.

## INTRODUCTION

Infectious diseases caused by arthropod-borne viruses (arboviruses) are on the rise worldwide, leading to several epidemics and pandemics in the last decades (1). An important family of arboviruses is the *Togaviridae*, including the genus *Alphavirus*. Their spherical enveloped virions have a size of about 70 nm in diameter, and the single-stranded positive-sense genome encodes for the non-structural proteins nsP1 and nsP4, the structural proteins E1, E2, E3, and 6K, and the capsid (2). Alphaviruses are zoonotic and can be transmitted within both sylvatic and urban cycles, involving virus-specific amplification hosts and vectors in each cycle. Human infection can result in two distinct types of disease: arthritic symptoms or encephalitic symptoms. Phylogenetic analysis based on the sequence of structural proteins shows distant clustering for most of the arthritogenic alphaviruses and the encephalitic alphaviruses (3). Human infection with arthritogenic alphaviruses can be asymptomatic or may lead to arthritic symptoms, including fever and rash. These symptoms typically last around a week, but some patients may suffer for several months from chronic conditions in the worst case (3–5). Infection with encephalitic alphaviruses ranges from asymptomatic outcomes to mild symptoms like febrile illness up to severe encephalitis (6).

The most important member of the arthritogenic alphaviruses is the chikungunya virus (CHIKV), which was first isolated in 1953, in today’s Tanzania (7). The sylvatic transmission cycle of CHIKV involves non-human primates and *Aedes* mosquitoes, resulting in sporadic and localized outbreaks in Africa over the past century. However, in the last 2 decades, CHIKV has become widespread in India, the Indian Ocean Islands, and the Americas, with huge outbreaks with millions of human infections caused by the establishment of an urban transmission cycle between humans and *Aedes* mosquitoes (3, 8). Local autochthonous outbreaks in Europe are also occurring more frequently (9). Human infection with CHIKV causes fever and severe arthralgia, which can become chronic for years (3). Another widespread member of the arthritogenic alphaviruses is the Sindbis virus (SINV), also first described in 1952 from Egypt (10). SINV transmission is maintained in an enzootic cycle between birds and mosquitoes. *Culex* mosquitoes are considered the primary vector (11). Whereas birds are the amplifying hosts, humans and other mammals are dead-end hosts (12). SINV is widely distributed in the Old World and Australia. Of the six different genotypes that are described, only lineage I is associated with human disease (13, 14). SINV lineage I is distributed to Africa, Europe, and the Middle East, and interestingly, there are only human cases described in South Africa and Fennoscandia (14). In the first days of infection, patients exhibit symptoms like rash, arthritis, and mild fever, but some also suffer from the long-term illness of arthralgia and myalgia for months or even years (15). The western equine encephalitis virus (WEEV) is an encephalitic alphavirus, first isolated in 1930 in California, USA (16). Genetic analysis showed that WEEV has arisen from a recombination of a Sindbis-like virus and eastern equine encephalitis virus (EEEV) (17). WEEV is only widespread in the Americas (18). Transmission occurs in an enzootic cycle between *Culex tarsalis* mosquitoes and birds or in an epizootic cycle between mammals and *Aedes* mosquitoes, whereas the *Aedes* mosquitoes act as a bridge vector, also infecting the dead-end hosts humans or equines (12, 19). Even as infection with WEEV in dead-end hosts is commonly inapparent, some patients develop a febrile illness or severe neuroinvasive disease with fatal outcomes. In comparison with humans, horses have a higher probability of severe disease progression (20).

The global rise of arbovirus infections and their spread into new, previously unaffected regions is driven by several factors, one of which is the presence of invasive mosquito species. Due to factors such as global trade and traveling, the increase in world population, land-use changes, and climate change, invasive mosquito species are establishing in new areas (1). Globally, the most important invasive mosquito is *Aedes albopictus*. Through this, the risk of arbovirus transmission can be increased in endemic areas, or new arboviruses can be introduced into non-endemic areas. In Europe, for example, *Ae. albopictus* is considered responsible for all cases of autochthonous CHIKV, Zika virus (ZIKV), and dengue virus (DENV) transmission (9). Besides the well-known invasive *Ae. albopictus*, there are other invasive mosquito species in Europe such as *Aedes japonicus* or, more specifically, one of the four subtypes: *Aedes japonicus japonicus* (21). In comparison to *Ae. albopictus*, these other invasive mosquito species are less studied, but might have the potential to boost arbovirus transmission in their new dispersal area. The first detection outside of its native geographic range was in the 1990s from the USA (22). Since the beginning of the 2000s, the invasion of Central Europe by *Ae. japonicus* took place. Currently, *Ae. japonicus* is documented in more than a dozen European countries, including Germany (23, 24). The opportunistic feeding pattern of *Ae. japonicus* with a slight preference for mammals, including humans, compared to birds makes this species a potential bridge vector for zoonotic arboviruses (25, 26). From experimental studies, it is known that *Ae. japonicus* is a potential vector for several flaviviruses, i.e., ZIKV, DENV, Usutu virus, and West Nile virus (27–30), as well as bunyaviruses, i.e., Rift Valley fever virus (31), and the roundworm *Dirofilaria* (32). Transmission potential for alphaviruses by *Ae. japonicus* is only known for two members of the genus: CHIKV and EEEV (27, 33).

Advanced technologies such as next-generation sequencing (NGS) and bioinformatic analysis have enabled a deeper understanding of the virome composition of mosquitoes, facilitating the discovery of intricate details about the interaction between virus infection and the mosquito’s antiviral immune response. Almost all examined mosquitoes contain insect-specific viruses (ISVs) (34). ISVs belong to various virus families and are defined as viruses that exclusively infect insects (35). However, the overall knowledge about ISVs and their influence on vector fitness or arbovirus infection/transmission is still very limited. A huge screening of field-collected *Aedes aegypti* mosquitoes from 12 different countries by Olmo et al. revealed Phasi Charoen-like virus (PCLV) and Humaita-Tubiacanga virus (HTV) as the most abundant ISVs in *Ae. aegypti* (36). Spatiotemporal as well as experimental analyses showed an increase in the probability of a DENV infection and transmission in mosquitoes that were also positive for HTV and PCLV, showing the influence of ISVs on arbovirus transmission (36). Studies on the virome of *Ae. japonicus* mosquitoes from the Netherlands and France revealed six insect-specific viruses (37). Moreover, *Ae. japonicus* narnavirus was also detected in *Ae. japonicus* specimens from the Netherlands, Japan, and the USA (30, 38, 39). The influence of these ISVs on arbovirus infection is still unknown. Another example is the ISV Wiesbaden virus (WBDV), recently discovered in *Aedes koreicus* from Germany (40). Association studies showed that coinfection of WBDV and CHIKV led to higher infection rates, while coinfection of WBDV and ZIKV showed no effect. WBDV was also detected in *Ae. japonicus* (40), but the effect on arbovirus infection in *Ae. japonicus* is also unknown.

To enhance our understanding of the vector competence of the invasive mosquito species *Ae. japonicus* for alphaviruses, we artificially infected field-caught *Ae. japonicus* mosquitoes from Germany with CHIKV, SINV, and WEEV. These viruses, which belong to both the arthritogenic and encephalitic clusters, are found in regions with either tropical or temperate climates. The infection rate, body titers, and transmission rate were analyzed in dependence on the influence of temperature and natural ISV infection on the vector competence of *Ae. japonicus*.

## MATERIALS AND METHODS

### Vector competence studies

#### Collection and rearing of mosquitoes

Eggs of *Ae. japonicus* were collected in the summer of 2019 and 2021 in southwestern Germany (49.524° N, 8.672° E/ 50.08° N, 8.17° E) via ovitraps. Rearing was performed in the insectary at 26°C, a relative humidity of 70%, and a light:dark photoperiod of 12:12, including 30 minutes of twilight. Eight percent fructose was available *ad libitum* on cotton pads. Morphological identification of larvae was performed by using the dichotomous key published in the “Guidelines for the Surveillance of Invasive Mosquitoes in Europe” (41). To be able to exclude natural virus infections that could affect the results, 10 randomly selected specimens were tested via pan-Alpha-, pan-Flavi-, and pan-Bunya-PCR (42–44), but all were negative.

#### Experimental infection of mosquitoes

Virus stocks of CHIKV (East-Central-South-African (ECSA) lineage, strain CNR_24/2014 EVAg, isolated 2014 from a human patient in France, seventh passage), SINV (lineage I, BNI-10865, GenBank MF 589985.1, isolated 2016 from a mosquito in Germany, sixth passage), and WEEV (strain 71V-1658, Genbank AF214040, isolated 1971 from a horse in Oregon, USA, unknown passage) (45) were propagated on Vero cells (*Chlorocebus sabaeus*; CVCL_0059, obtained from ATCC, cat # CCL-81) with growth medium (Dulbecco’s modified eagle medium, supplemented with 3% filtrated bovine serum [FBS], 100 mg/mL streptomycin, 100 U/mL penicillin, 1% non-essential amino acids, 1% sodium pyruvate). Ten- to 31-day-old females were sorted into vials and starved overnight. Blood meals were offered for 2–4 hours in two 50 µL drops on the bottom of the vials, containing 50% human blood (blood group 0, expired blood bags from a local blood bank; blood donor samples are anonymized, i.e., ethical approval is not required), 30% of an 8% fructose solution, 10% FBS, and 10% virus stock. The final virus concentration was set to 10^7^ plaque-forming units per milliliter. The overall feeding rate (number of fed females per total females) was 41% (461/1,113). After the blood meal, fully engorged females were sorted into new vials and incubated for 7 or 14 days with a relative humidity of 70% and a 12:12 photoperiod at either 21°C ± 5°C, 24°C ± 5°C, or 27°C ± 5°C. Fluctuation was set to mimic the day-night rhythm. Fructose solution was available *ad libitum* on a cotton pad. All combinations of temperature/virus/incubation time were repeated at least two times. Vector competence studies with CHIKV were performed in 2019, with SINV in 2019/2021, and with WEEV in 2021.

#### Infection analysis

To test the transmission of infectious virus particles, a salivation assay was performed as previously described (46). Briefly, 20 µL phosphate-buffered saline containing the saliva was added to Vero cells in a 96-well plate with 200 µL growth medium per well, containing an additional 5 µg/mL amphotericin B. Cells were incubated at 37°C and 5% CO_2_ for 5 days. For saliva-infected cells with cytopathic effect, the supernatant was collected, RNA was extracted (QIAamp Viral RNA Mini Kit, Qiagen, Hilden, Germany), and CHIKV-, SINV-, or WEEV-specific quantitative reverse transcription Polymerase chain reaction (qRT-PCR) was performed to make sure CPE was induced by viruses and not by contamination. The following qRT-PCR protocols were used: RealStar Chikungunya RT-PCR Kit 2.0 (altona Diagnostics, Hamburg, Germany), SINV qRT-PCR as described by Jansen et al. (11), and WEEV qRT-PCR as described by Lambert et al. (WEE TaqMan primer and probe of Lambert et al., using the following standard: “atgctgaaagtcggcctgcgtatagtatacggcaacaccaccgcgcacctggatacgttcgtcaatggcgcta”) (47), with addition of the VetMAX Xeno Internal Positive Control (Applied Biosystems, Thermo Fisher Scientific Corporation, Waltham, MA, USA). A calculation of the infection rate (IR, number of virus-RNA-positive females per number of fed females) and transmission rate (TR, number of virus-positive saliva per number of virus-positive bodies) was conducted. Additionally, the mean body virus titer was determined by analyzing the mosquito bodies. Whole mosquito bodies, including the head, were homogenized. RNA was extracted and analyzed via qRT-PCR as described above.

### Virome characterization of field-caught and laboratory-reared *Ae. japonicus* from Germany

#### Metagenomic sequencing of the mosquito samples

A total of 30 *Ae. japonicus* female mosquitoes collected in 2019 were combined into five pools and placed into 2 mL secure-lock tubes with zirconia beads (2 mm, BioSpec Products, Bartlesville, OK, USA) along with 0.7 mL of chilled Dulbecco’s modified Eagle’s medium (Sigma-Aldrich, St. Louis, MO, USA). The mosquitoes were homogenized using a TissueLyser II (Qiagen, Hilden, Germany) for 2 minutes at a frequency of 30 Hz–50 Hz. Subsequently, the suspension was clarified by centrifugation for 1 minute at 8,000 rpm and 4°C followed by RNA extraction using the QIAamp Viral RNA Mini Kit (Qiagen, Hilden, Germany) following the manufacturer’s instructions. The isolated RNA was subjected to metagenomic next-generation sequencing as described elsewhere (48). In brief, following random RT-PCR amplification of the RNA, the isolated viral RNA underwent library preparation using a QIAseq FX DNA Library Kit (Qiagen, Hilden, Germany). Normalized samples were combined and sequenced using 2 × 150 base pairs (bp) paired-end NextSeq 550 reagent kits v.2.5 (Illumina, San Diego, CA, USA) on a NextSeq 550 platform.

#### Virome characterization of *Ae. japonicus* and phylogeny

The initial quality assessment of the generated raw reads involved trimming and filtering reads with Phred quality scores <20 to eliminate polyclonal and low-quality reads (<55 bases long) utilizing CLC Workbench (Qiagen, Hilden, Germany). Subsequently, the filtered raw reads were *de novo* assembled using Trinity v.2.6.64239 and CLC Workbench. Comparative analysis was conducted by matching against a nonredundant viral proteome database (NCBI) through BLASTx with an E-value cutoff of 0.001. Further comparison of virus-like contigs was performed against all protein sequences in nonredundant protein databases with a default E-value cutoff of 0.001. The output from viral metagenomics and metatranscriptomics was visualized and analyzed utilizing MEGAN (49). Sequence analysis, genome assembly, genomic organization, and multiple alignments of the detected viruses were executed using Geneious Prime (Biomatters, Auckland, New Zealand). Amino acid sequences of complete or partial genomes of the newly identified virus species were aligned with closely related reference viruses of the respective virus family or species. Alignment of amino acid sequences was performed using the E-INS-i algorithm implemented in MAFFT. The evolutionary relationships of the detected viruses were assessed by constructing phylogenetic trees utilizing the maximum-likelihood method in Seaview (50), incorporating subtree pruning and regrafting branch swapping, and an approximate likelihood ratio test for specific node support evaluation. All sequences of the identified ISVs were uploaded to GenBank (accession no. PP706076–PP706081).

#### Detection of novel insect-specific viruses

Detection of the identified novel insect-specific viruses was performed via RT-PCR for all specimens, which were investigated in the vector competence studies. All RT-PCRs were performed by using the Superscript III one-step RT-PCR Kit (Invitrogen, Carlsbad, CA, USA). RT-PCR was performed as follows: reverse transcription at 60°C for 1 minute, 50°C for 45 minutes, PCR cycling with initial 94°C for 2 minutes and 45 cycles of 94°C for 15 seconds, 55°C for 30 seconds, 68°C for 30 seconds, and a final extension of 68°C for 7 minutes. Specific primers are listed in Table 1. Another ISV, WBDV, was detected in *Ae. japonicus* from the same location in previous studies (40). We screened the investigated specimens additionally for WBDV.

**TABLE 1:** List of discovered *Ae. japonicus*-associated viruses, specific primers, and PCR product size^*a*^

Insect-specific virus	Forward primer	Reverse primer	Product size (bp)
*Aedes japonicus* negevirus	TCGTATCCTACCTTGTCACC	GGTCATCAACAAGCAGCATA	275
*Aedes japonicus* anphevirus	GCTAAATGCAGACCTGTTCC	CCCTCCTGTTATTCCACTCA	290
*Aedes japonicus* narnavirus	GGGAACTCCTATGTACAAGG	CATTCGAGCTGTGTCAGTAG	279
*Aedes japonicus* chuvirus	CATCTACCAGCATCCACTTG	TTGGAGAGCACGGAGTATTG	205
*Aedes japonicus* bunyavirus 2	TGGCTCTTTTACTGGTAGGC	GACCTCCACCTTTCCTTAATC	269
*Aedes japonicus* partiti-like virus	GACCTCCACTCCTTTTGTGT	CTCCACGTTCCTTACTCAAC	230

^*a*^ bp = base pairs.

### Statistical analysis of coinfection

Statistical analysis of coinfection was performed in R (version 4.2.2) using the R-Studio IDE (version 2022.12.0) (51). Chi-square test was used in bivariate analysis to determine the association of each ISV and each arbovirus. The link between ISV infection status and arbovirus body titer was analyzed using individual binomial generalized linear models per combination of arbovirus and ISV. The infection status of ISVs was used as the response variable and arbovirus body titer as the explanatory variable.

## RESULTS

### Vector competence studies

Transmission of all three alphaviruses (CHIKV, SINV, WEEV) by *Ae. japonicus* was observed (Table 2). Transmission of CHIKV was only observed at the highest temperature profile of 27°C ± 5°C, with a TR of 2.9%. IRs at all three investigated temperature profiles were in a similar range (81.8% at 21°C ± 5°C, 81.6% at 24°C ± 5°C, 89.5% at 27°C ± 5°C) (Table 2). The mean body titer increased with temperature from a mean 3.2 (95% CI: 2.5–3.9) to 5.0 (4.2–5.8) log10 RNA copies per specimen.

**TABLE 2:** Vector competence studies with *Aedes japonicus japonicus* and three different alphaviruses at two/three different temperature profiles^*a*^

Virus	dpi	Temperature (°C)	*n*	IR (%)	Mean (95% CI) log10 RNA copies per specimen	TR (%)
Chikungunya virus	14	21 +/- 5	33	81.8 (27/33)	3.2 (2.5–3.9)	0.0 (0/33)
	14	24 +/- 5	38	81.6 (31/38)	3.6 (2.9–4.3)	0.0 (0/31)
	14	27 +/- 5	38	89.5 (34/38)	5.0 (4.2–5.8)	2.9 (1/34)
Sindbis virus	14	21 +/- 5	40	82.0 (32/39)	5.5 (4.3–6.7)	43.8 (14/32)
	14	27 +/- 5	28	100.0 (28/28)	6.0 (5.0–6.9)	57.1 (16/28)
Western equine encephalitis virus	7	21 +/- 5	34	100.0 (34/34)	6.9 (6.3–7.5)	32.4 (11/34)
	7	27 +/- 5	28	100.0 (28/28)	6.8 (6.2–7.2)	32.1 (9/28)

^*a*^ dpi, days post-infection; *n*, number of investigated specimens.

For SINV, transmission could be observed at both temperature profiles (Table 2). At 21°C ± 5°C, the IR was 82.0%. At 27°C±5°C, the IR increased up to 100%. The TR increased from 43.8% to 57.1%, respectively. As for CHIKV, the mean body titer increased from 5.5 log10 RNA copies per specimen (4.3–6.7) at 21°C ± 5°C to 6.0 (5.0–6.9) log10 RNA copies per specimen at 27°C ± 5°C.

WEEV transmission was the same at both investigated temperatures (21°C ± 5°C and 27°C ± 5°C), with an IR of 100% and a TR of 32% for both analyzed temperature profiles (Table 2). The mean body titer was also very similar at both temperature profiles with a mean of 6.9 (6.3–7.5) at 21°C ± 5°C and 6.8 (6.2–7.2) log10 RNA copies per specimen at 27°C ± 5°C.

### Identification of *Aedes japonicus*-associated viruses

**Fig 1:**
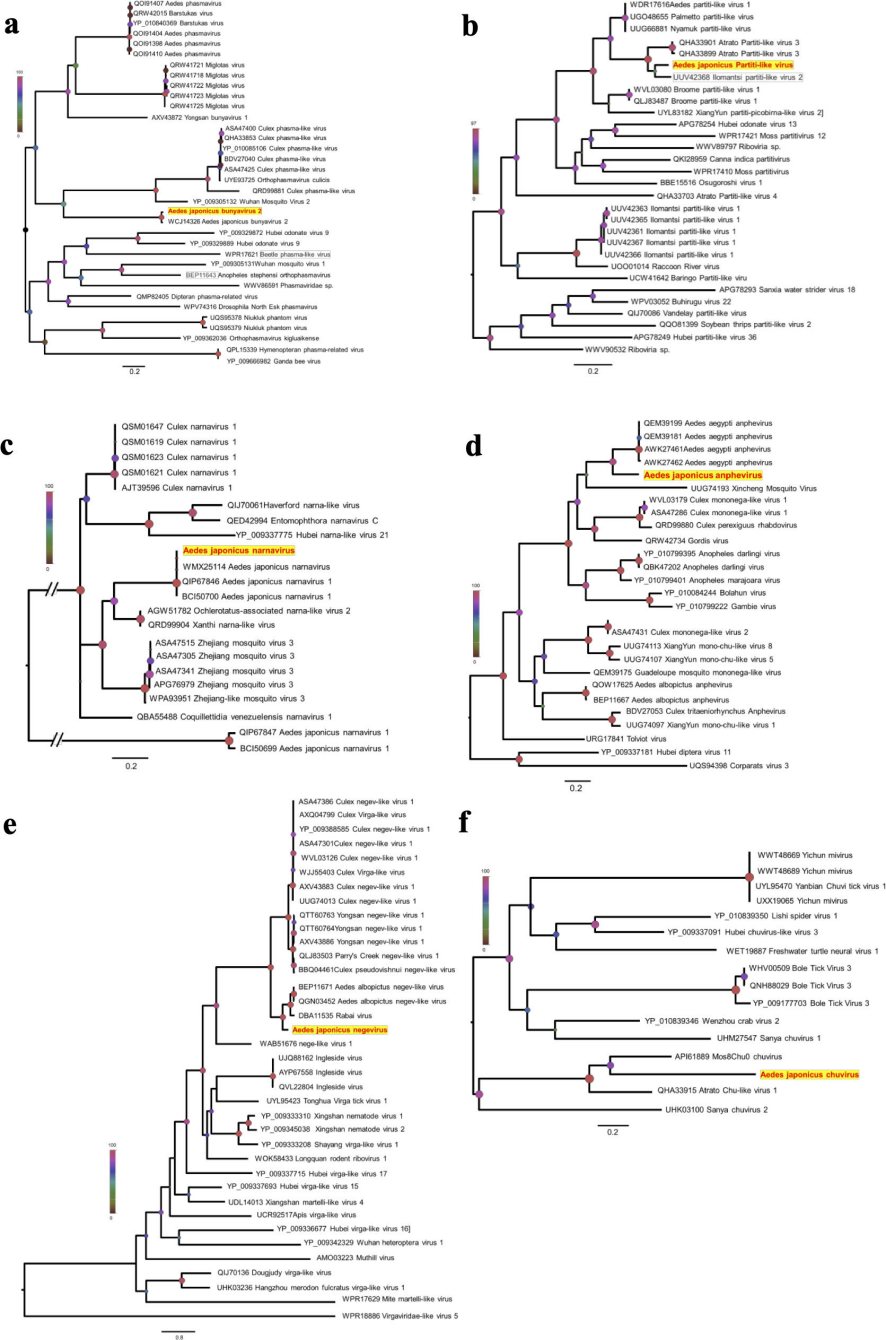
The maximum-likelihood phylogenetic trees show the evolutionary relationship of the viruses identified in *Ae. japonicus* mosquitoes from this study (bolded in red in the context of representatives of their closest relatives). Phylogenetic trees were generated using the multiple sequence alignments of glycoprotein (**a, **d), RNA-dependent RNA polymerase (RdRp) (**b, c, **e), and nucleocapsid (f) amino acid sequences. Consensus maximum-likelihood trees were performed under the best-fit amino acid substitution models identified LG+G+I+F (a; *Aedes japonicus* bunyavirus 2; *Phasmaviridae*), LG+G+I (b; *Aedes japonicus* partiti-like virus; *Partitiviridae*), WAG+G (c; *Aedes japonicus* narnavirus; *Narnaviridae*), LG+I+F (d; *Aedes japonicus* anphevirus; *Xinmoviridae*), LG+G+F (e; *Aedes japonicus* negevirus; *Phasmaviridae*), and LG+G+I+F (f; *Aedes japonicus* chuvirus; *Chuviridae*) using Akaike information criterion as the model selection framework in jModelTest (52). To assess the robustness of each node, a bootstrap resampling process was performed (1,000 replicates), again using the Nearest-Neighbour interchange (NNI) branch swapping method available in Seaview. All trees are midpoint-rooted and scaled with the branch length indicating the number of amino acid substitutions per site. Node circles represent the bootstrap values as shown in the legend. Accession numbers for the protein sequences are shown with the virus names.

We characterized the total transcriptome of five mosquito pools, representing 30 specimens of *Ae. japonicus* sampled in Germany. Subsequent BLAST analyses of the contigs revealed the presence of several RNA viral species that fell into a wide range of RNA virus families. We identified several putative negative- and positive-stranded RNA viruses from the *Phasmaviridae* family (*Aedes japonicus* bunyavirus 2, accession no. PP706080), *Partitiviridae* (*Aedes japonicus* partiti-like virus, accession no. PP706077), *Narnaviridae* (*Aedes japonicus* narnavirus, accession no. PP706079), *Xinmoviridae* (*Aedes japonicus* anphevirus, accession no. PP706076), *Negevirus* (*Aedes japonicus* negevirus, accession no. PP706081), and *Chuviridae* (*Aedes japonicus* chuvirus, accession no. PP706078) (Fig. 1).

BLASTp and the subsequent phylogenetic examination of newly predicted virus sequences, particularly focusing on RNA-dependent RNA polymerase (RdRp) or other genes, suggest that the viruses described in this study share close relationships with viruses previously identified in mosquitoes, dipterans, or other related arthropods known to infect mosquitoes or other insect species (Fig. 1).

### Correlation of arbovirus infection with insect-specific viruses

The overall infection rate for the detected ISVs in all investigated specimens was 28.5% (68/238) for *Aedes japonicus* negevirus, 85.7% (204/238) for *Aedes japonicus* anphevirus, 96.2% (229/238) for *Aedes japonicus* narnavirus, 97.0% (231/238) for *Aedes japonicus* chuvirus, 68.9% (164/238) for *Aedes japonicus* bunyavirus 2, 5.0% (12/238) for *Aedes japonicus* partiti-like virus, and 23.1% (55/238) for WBDV (Table 3).

**TABLE 3:** Infection and coinfection rate (%) for seven insect-specific viruses in *Ae. japonicus japonicus* specimens investigated in vector competence studies^*a*^

Alpha virus	dpi	Temperature (°C)	*Aedes japonicus* negevirus			*Aedes japonicus* anphevirus			*Aedes japonicus* narnavirus			*Aedes japonicus* chuvirus		
			IR	CR_body_	CR_saliva_	IR	CR_body_	CR_saliva_	IR	CR_body_	CR_saliva_	IR	CR_body_	CR_saliva_
CHIKV	14	21 ± 5	3.0 (1/33)	3.0 (1/33)	0 (0/33)	93.9 (31/33)	75.8 (25/33)	0 (0/33)	100.0 (33/33)	81.8 (27/33)	0 (0/33)	100.0 (33/33)	81.8 (27/33)	0 (0/33)
	14	24 ± 5	2.6 (1/38)	2.6 (1/38)	0 (0/38)	94.7 (36/38)	76.3 (29/38)	0 (0/38)	97.4 (37/38)	78.9 (30/38)	0 (0/38)	100 (38/38)	81.6 (31/38)	0 (0/38)
	14	27 ± 5	10.5 (4/38)	10.5 (4/38)	0 (0/38)	94.7 (36/38)	84.2 (32/38)	2.6 (1/38)	97.4 (37/38)	86.8 (33/38)	2.6 (1/38)	100.0 (38/38)	89.5 (34/38)	2.6 (1/38)
SINV	14	21 ± 5	53.8 (21/39)	38.5 (15/39)	20.5 (8/39)	89.7 (35/39)	71.8 (28/39)	30.8 (12/39)	100 (39/39)	79.5 (31/39)	35.9 (14/39)	100.0 (39/39)	79.5 (31/39)	35.9 (14/39)
	14	27 ± 5	28.6 (8/28)	28.6 (8/28)	21.4 (6/28)	78.6 (22/28)	78.6 (22/28)	50 (14/28)	100.0 (28/28)	100.0 (28/28)	57.1 (16/28)	100.0 (28/28)	100.0 (28/28)	57.1 (16/28)
WEEV	7	21 ± 5	26.5 (9/34)	26.5 (9/34)	8.8 (3/34)	58.8 (20/34)	58.8 (20/34)	20.6 (7/34)	91.2 (31/34)	91.2 (31/34)	32.4 (11/34)	79.4 (27/34)	79.4 (27/34)	26.5 (9/34)
	7	27 ± 5	85.7 (24/28)	85.7 (24/28)	25 (7/28)	85.7 (24/28)	85.7 (24/28)	25 (7/28)	85.7 (24/28)	85.7 (24/28)	25 (7/28)	100.0 (28/28)	100.0 (28/28)	32.1 (9/28)
Total			28.5 (68/238)			85.7 (204/238)			96.2 229/238			97.0 (231/238)		

	dpi	Temperature (°C)	*Aedes japonicus* bunyavirus 2			*Aedes japonicus* partiti- like virus			Wiesbaden virus		
			IR	CR_body_	CR_saliva_	IR	CR_body_	CR_saliva_	IR	CR_body_	CR_saliva_
CHIKV	14	21 ± 5	48.5 (16/33)	42.4 (14/33)	0 (0/33)	6.1 (2/33)	6.1 (2/33)	0 (0/33)	0.0 (0/33)	0.0 (0/33)	0 (0/33)
	14	24 ± 5	81.6 (31/38)	65.8 (25/38)	0 (0/38)	2.6 (1/38)	2.6 (1/38)	0 (0/38)	0.0 (0/38)	0.0 (0/38)	0 (0/38)
	14	27 ± 5	73.7 (28/38)	68.4 (26/38)	2.6 (1/38)	0.0 (0/38)	0.0 (0/38)	0 (0/38)	0.0 (0/38)	0.0 (0/38)	0 (0/38)
SINV	14	21 ± 5	74.4 (29/39)	59.0 (23/39)	30.8 (12/39)	15.4 (6/39)	15.4 (6/39)	5.1 (2/39)	41.0 (16/40)	30.8 (12/39)	15.4 (6/39)
	14	27 ± 5	78.6 (22/28)	78.6 (22/28)	53.6 (15/28)	3.6 (1/28)	3.6 (1/28)	3.6 (1/28)	32.1 (9/28)	32.1 (9/28)	7.1 (2/28)
WEEV	7	21 ± 5	52.9 (18/34)	52.9 (18/34)	14.7 (5/34)	0.0 (0/34)	0.0 (0/34)	0 (0/34)	58.8 (20/34)	58.8 (20/34)	14.7 (5/34)
	7	27 ± 5	71.4 (20/28)	71.4 (20/28)	21.4 (6/28)	7.1 (2/28)	7.1 (2/28)	0 (0/28)	35.7 (10/28)	35.7 (10/28)	14.3 (4/28)
Total			68.9 (164/238)			5.0 (12/238)			23.1 (55/238)		

^*a*^ dpi, days post-infection; CR_body_, coinfection rate of ISV-positive body and alphavirus-positive body; CR_saliva_, coinfection rate of ISV-positive body and alphavirus-positive saliva.

**Fig 2:**
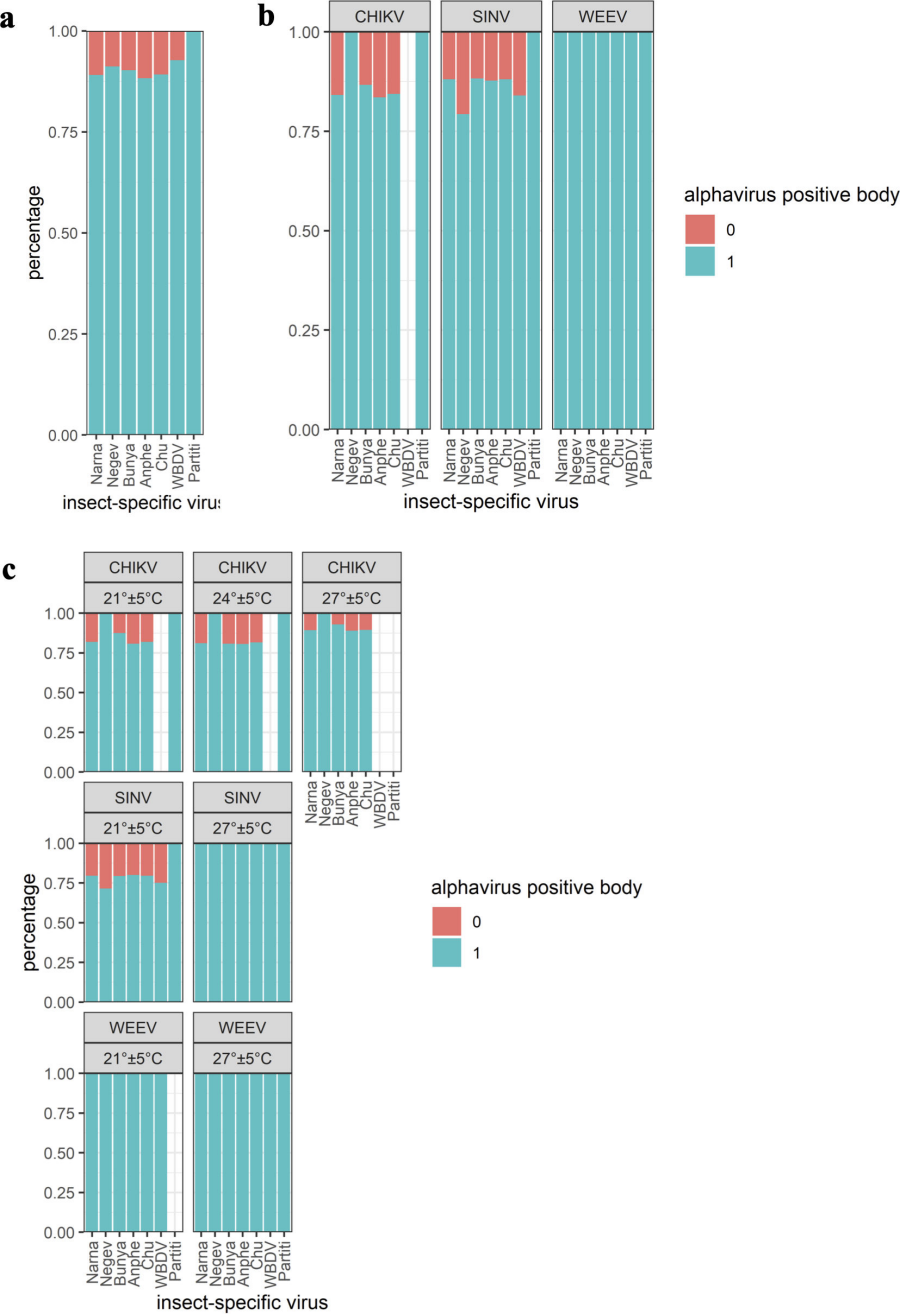
(a) Percentage of alphavirus-positive/negative specimens per ISV; (b) percentage of CHIKV/SINV/WEEV-positive specimens per ISV; (c) percentage of CHIKV/SINV/WEEV-positive specimens per ISV and temperature. Turquoise: alpha-positive body, pale pink: alpha-negative body. Anphe, *Aedes japonicus* anphevirus; Bunya, *Aedes japonicus* bunyavirus 2; Chu, *Aedes japonicus* chuvirus; Narna, *Aedes japonicus* narnavirus; Negev, *Aedes japonicus* negevirus; Partiti, *Aedes japonicus* partiti-like virus.

The majority of ISV-positive bodies were also positive for an arbovirus (Table 3; Fig. 2a). All combinations of co- and multiple infections between the seven ISVs and the three arboviruses were detected, except CHIKV-positive bodies were all negative for WBDV infection at all investigated temperatures. Furthermore, no *Aedes japonicus* partiti-like virus coinfection was detected for CHIKV-infected specimens at 27°C ± 5°C and WEEV-infected specimens at 21°C ± 5°C (Fig. 2b + Fig. 2c).

In general, an underlying ISV infection did not have a statistically significant impact on the arbovirus IR, whether analyzing each arbovirus IR individually or by combining all arbovirus IRs (Fig. 2; Table S1A and B). Investigating the impact of ISV infection on the mean body arbovirus titer, *Ae. japonicus* specimens showed a statistically significant higher titer for specimens coinfected with *Aedes japonicus* negevirus and WBDV, while no effect on body titer was detected for all other ISVs (Fig. S1a; Table S1C). Looking in more detail separately for each of the three different arboviruses, no impact of ISVs on the arbovirus titer was observed for CHIKV and SINV (Fig. S1b; Table S1D). Only specimens infected with WEEV showed a statistically significant higher titer if coinfected with *Aedes japonicus* anphevirus, *Aedes japonicus* chuvirus, or *Aedes japonicus* bunyavirus 2 (Fig. S1b and c; Table S1D).

**Fig 3:**
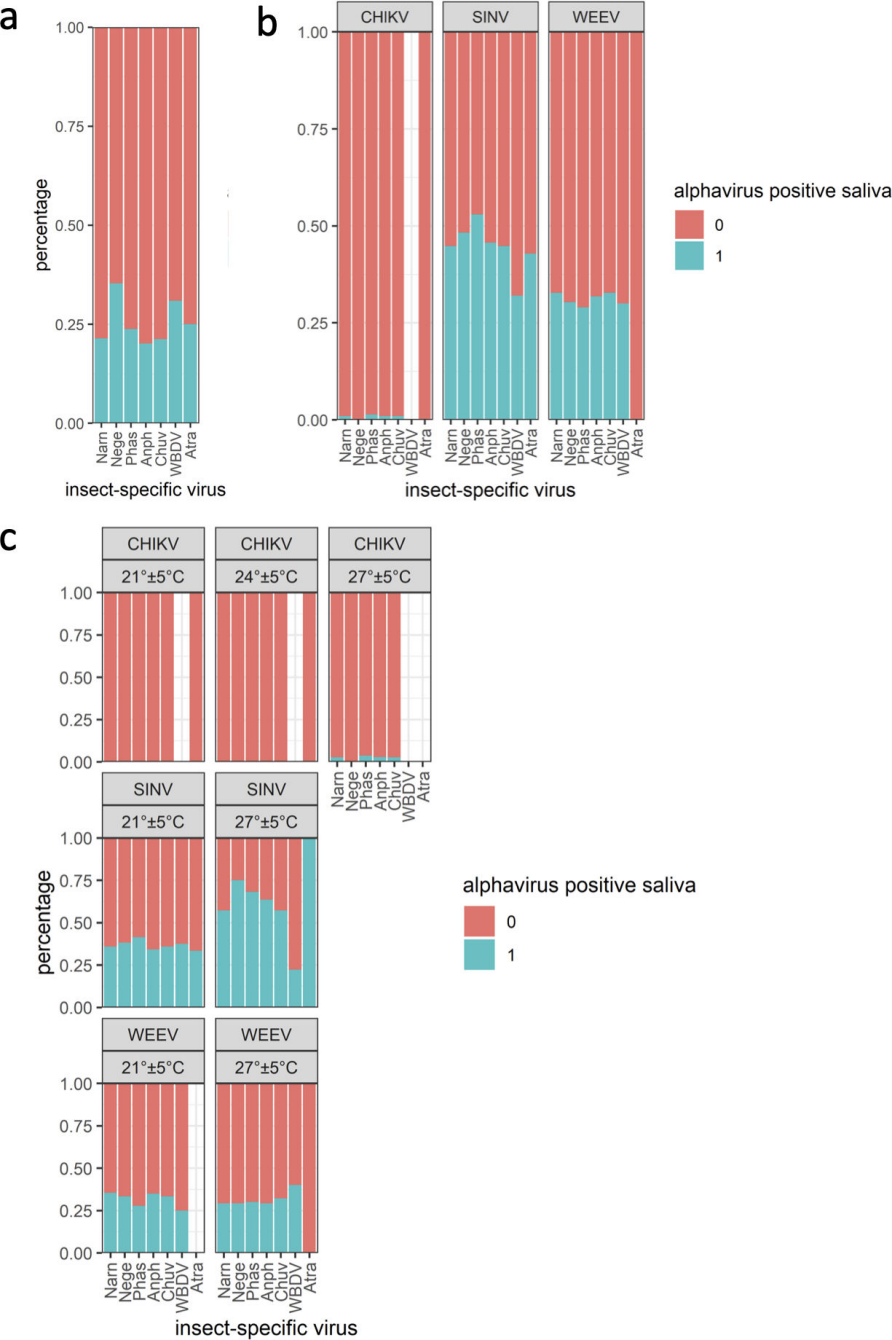
(a) Percentage of alphavirus-positive/negative saliva per ISV; (b) percentage of CHIKV/SINV/WEEV-positive saliva per ISV; (c) percentage of CHIKV/SINV/WEEV-positive saliva per ISV and temperature. Turquoise: alpha-positive saliva, pale pink: alpha-negative saliva. Anphe, *Aedes japonicus* anphevirus; Bunya, *Aedes japonicus* bunyavirus 2; Chu, *Aedes japonicus* chuvirus; Narna, *Aedes japonicus* narnavirus; Negev, *Aedes japonicus* negevirus; Partiti, *Aedes japonicus* partiti-like virus.

There was no statistically significant impact on the overall arbovirus TR due to coinfection with an ISV; only specimens coinfected with *Aedes japonicus* negevirus had a significantly higher TR for arboviruses (Fig. 3a; Table S1A). Looking individually for the three arboviruses, no significant impact of ISVs on any arbovirus TR was observed. Only specimens infected with *Aedes japonicus* bunyavirus 2 showed a statistically significant higher TR for SINV (Fig. 3b and c; Table S1B).

## DISCUSSION

*Aedes japonicus* can transmit all three investigated alphaviruses: CHIKV, SINV, and WEEV. Our results for CHIKV are in line with the results of Schaffner et al., who experimentally proved CHIKV transmission by *Ae. japonicus* mosquitoes from Switzerland at 28°C (27). The detected TR of Schaffner et al. with 13.3% was higher compared to our findings of 2.9%. The deviating values of Schaffner et al. could be caused by (i) a different virus strain that was used in their studies (even if it also belongs to the ECSA lineage) or (ii) the most important difference: a different *Ae. japonicus* population (field collected in 2010, from Switzerland), whose specific microbiome, and particularly the virome, influences their vector competence. Such differences in vector competence are also shown by Gloria-Soria et al. for different *Ae. albopictus* populations infected with different CHIKV strains of the USA (53). The detected IR of CHIKV was around 80% for all three temperature profiles, but looking through the body titer, there were differences. At the highest temperature profile of 27°C ± 5°C, the mean body titer was 5.0 log10 RNA copies per specimen, and transmission was observed. For both lower temperature profiles, no transmission was observed, and the mean body titer was lower, with a mean titer of 3.2 or 3.6 log10 RNA copies per specimen. A specific viral load in the body may be required for the virus to overcome the salivary gland barrier; however, further research is needed to confirm this.

Transmission of SINV and WEEV was observed for all investigated temperature conditions, and the mean body titer amounted to between 5.5 to 6.9 log10 RNA copies per specimen. Whereby the transmission of CHIKV was clearly temperature-dependent, transmission of SINV and WEEV did not show such pronounced differences between the investigated temperature profiles. Temperature-dependent transmission of arboviruses has been observed across various arboviruses and mosquito species, but it appears to be highly specific to each virus-mosquito species combination. For instance, transmission of CHIKV by *Ae. koreicus* is temperature-dependent, whereas transmission of the same CHIKV strain by *Ae. albopictus* does not exhibit a clear temperature dependence (40, 54, 55).

The low TR for CHIKV, observed only at the highest temperature, suggests a relatively low risk of CHIKV transmission by *Ae. japonicus*. This is particularly relevant given that CHIKV is endemic in tropical regions, while the distribution of *Ae. japonicus* is restricted to temperate regions. In contrast, high TRs were detected for SINV and WEEV at 21°±5°C and 27°±5°C and since *Ae. japonicus* is found in areas where these viruses are endemic in Europe or America, the results highlight the potential role of *Ae. japonicus* as a bridge vector for both viruses.

Vector competence is not only influenced by abiotic factors like temperature; it is also affected by biotic factors like the virome. Studies by Shi et al. revealed a stable core virome of lab- and field-collected *Ae. albopictus* mosquitoes, but only in field-caught *Ae. albopictus did* they additionally find an environment-derived core virome (56). This highlights the advantage of using field-caught mosquitoes for vector competence studies. Three of the detected ISVs were also detected in *Ae. japonicus* from the Netherlands and France by Abbo et al.: *Aedes japonicus* anphevirus 1, *Aedes japonicus* bunyavirus 2, and *Aedes japonicus* narnavirus (37). So far, all investigations of the virome of *Ae. japonicus* from Europe, Asia, and America detected *Aedes japonicus* narnavirus (30, 38, 39), which may implicate an important role of this ISV for *Ae. japonicus* mosquitoes. Just as in previous studies of *Ae. japonicus* from the same location, WBDV could be detected, supporting the hypothesis that WBDV might not be species- but habitat-specific (57). The phylogenetic analysis of the newly identified ISVs in this study, *Aedes japonicus* negevirus, *Aedes japonicus* chuvirus, *Aedes japonicus* partiti-like virus (Fig. 1), indicates the clustering of viruses associated with mosquitoes from diverse geographic regions or mosquito species, hinting at prolonged interactions between these viruses and their mosquito hosts. Notably, these clusters often contain multiple viral lineages linked to single or multiple host species or genera, indicating a nuanced pattern of virus-host relationships without clear evidence of virus-host codivergence.

We detected coinfection of the seven investigated ISVs with all arboviruses, except for coinfection of CHIKV and WBDV. Interestingly, in previous studies, coinfection of CHIKV and WBDV was detected in *Ae. koreicus* (40). This gives a hint that there are possible differences in these closely related species, which might be due to different genetic features or eco-immunological factors shaping the immune reaction toward specific ISVs (58).

Given the prevalence of multi-infections involving various ISVs and the three alphaviruses, it is clear that the interaction between ISVs and arboviruses under natural conditions is complex and multidimensional. Predicting these interactions is challenging without extensive sample sizes, which are necessary to elucidate the varying outcomes observed in different studies (36). This complex system might get even more complicated when players beside the ISVs from the natural bacterial microbiome like Wolbachia are also considered (59).

### Conclusion

Invasive mosquito species can have a huge impact on the spread of arboviruses. Data from future climate models predict the further spread of *Ae. japonicus* in the northern hemisphere (60), indicating the importance of gaining better knowledge about the risk of arbovirus transmission by *Ae. japonicus*. In this study, we could show experimental transmission of CHIKV, SINV, and WEEV by *Ae. japonicus*. Low transmission of CHIKV was exclusively observed at the high-temperature profile, while transmission of SINV and WEEV was observed at all investigated temperature profiles with higher transmission rates of 32%–57%. Multi-infections with ISVs are very common and can have an impact on arbovirus transmission, either by blocking or boosting transmission. However, the interplay between arbovirus infections and ISV infections is highly complex, and so far, the mechanisms are not fully understood.

## SUPPLEMENTAL MATERIAL

Figure S1 spectrum.02668-24-s0001.tiff Correlation of mean body alphavirus titer per ISV.

Supplemental legend spectrum.02668-24-s0002.docx Legend for Fig. S1.

Table S1 spectrum.02668-24-s0003.xlsx Interaction of arboviruses and insect-specific viruses.

ASM does not own the copyrights to Supplemental Material that may be linked to, or accessed through, an article. The authors have granted ASM a non-exclusive, world-wide license to publish the Supplemental Material files. Please contact the corresponding author directly for reuse.
