# Supplementary figures and images for: The impact of temperature and insect-specific viruses on the transmission of alphaviruses by Aedes japonicus japonicus
Source: Microbiol Spectr. 2025 Apr 30;13(6):e02668-24. doi: 10.1128/spectrum.02668-24 (PMC12131847; doi:10.1128/spectrum.02668-24)

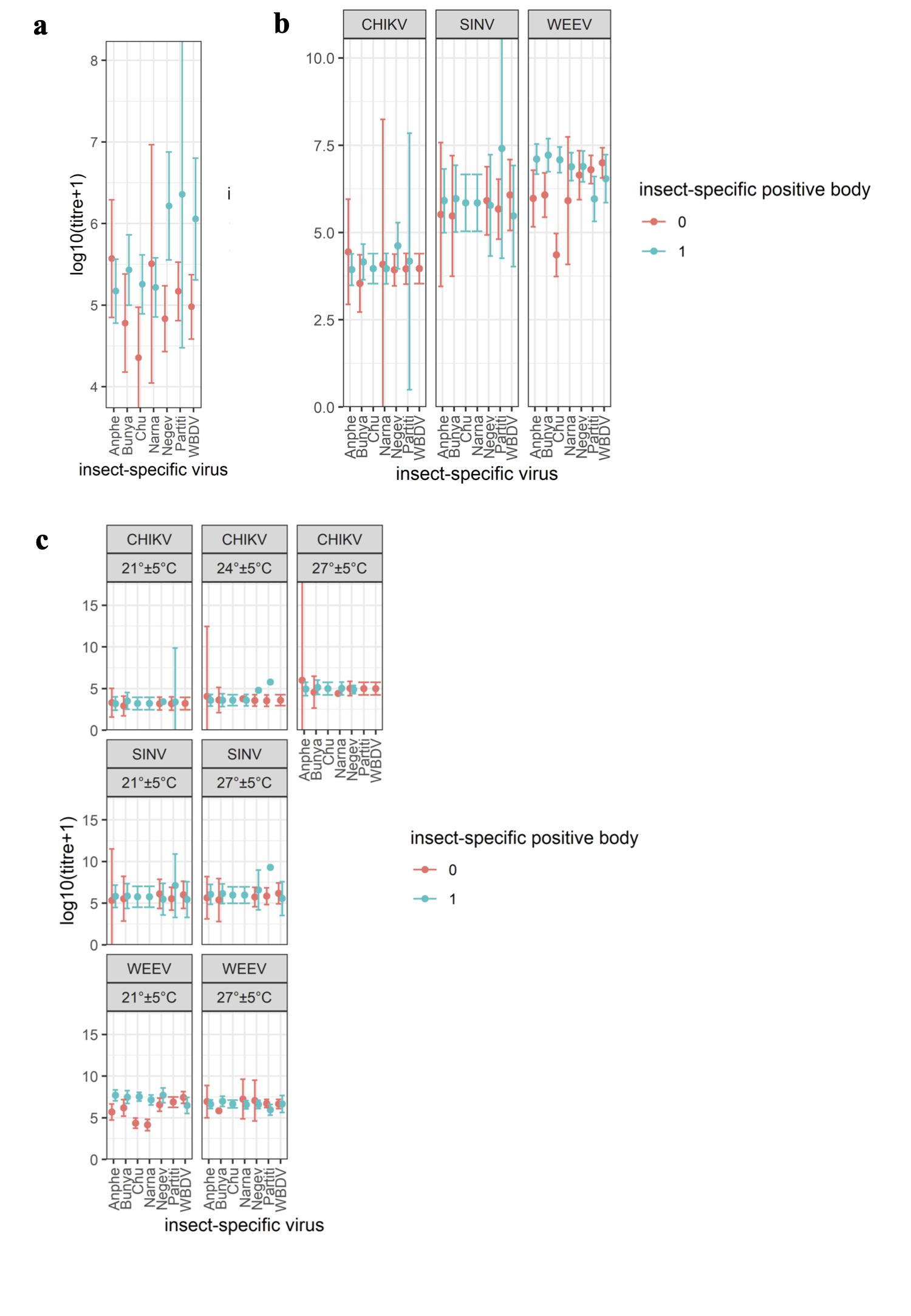

Supplement: Figure S1 — Correlation of mean body alphavirus titer per ISV. [file spectrum.02668-24-s0001.tiff]
