# Supplementary material for: The impact of temperature and insect-specific viruses on the transmission of alphaviruses by Aedes japonicus japonicus
Source: Microbiol Spectr. 2025 Apr 30;13(6):e02668-24. doi: 10.1128/spectrum.02668-24 (PMC12131847; doi:10.1128/spectrum.02668-24)
Supplement: Supplemental legend — Legend for Fig. S1. [file spectrum.02668-24-s0002.docx]

**Supplementary figure 1**: **(a)** mean body titer of alphavirus per ISV; **(b)** mean body titer of CHIKV/SINV/WEEV-positive saliva per ISV; **(c)** mean body titer of CHIKV/SINV/WEEV-positive saliva per ISV and temperature. turquoise: ISV positive body, pale pink: ISV negative body. Anphe: Aedes japonicus anphevirus, Bunya: Aedes japonicus bunyavirus 2, Chu: Aedes japonicus chuvirus, Narna: Aedes japonicus narnavirus, Negev: Aedes japonicus negevirus, Partiti: Aedes japonicus Partiti-like virus.
